# Cancer stem cell targeted therapy: progress amid controversies

**DOI:** 10.18632/oncotarget.6176

**Published:** 2015-10-19

**Authors:** Tao Wang, Sarah Shigdar, Michael P. Gantier, Yingchun Hou, Li Wang, Yong Li, Hadi Al Shamaileh, Wang Yin, Shu-Feng Zhou, Xinhan Zhao, Wei Duan

**Affiliations:** ^1^ School of Nursing, Zhengzhou University, Zhengzhou, China; ^2^ School of Medicine, Deakin University, Waurn Ponds, Victoria, Australia; ^3^ Centre for Cancer Research, Hudson Institute of Medical Research, Clayton, Victoria, Australia; ^4^ Department of Molecular and Translational Science, Monash University, Clayton, Victoria, Australia; ^5^ Co-Innovation Center for Qinba Region's Sustainable Development, Shaanxi Normal University, Xi'an, China; ^6^ Department of Gynecologic Oncology, Henan Cancer Hospital, The Affiliated Cancer Hospital of Zhengzhou University, Zhengzhou, China; ^7^ Cancer Care Centre, St George Hospital and St George Clinical School, University of New South Wales (UNSW), Kensington, Australia; ^8^ Department of Pharmaceutical Sciences, College of Pharmacy, University of South Florida, Tampa, FL, USA; ^9^ Department of Medical Oncology, The First Affiliated Hospital of Xi'an Jiaotong University School of Medicine, Xi'an, China

**Keywords:** cancer, cancer stem cell, anti-cancer treatment, cancer stem cell marker, cancer stem cell model

## Abstract

Although cancer stem cells have been well characterized in numerous malignancies, the fundamental characteristics of this group of cells, however, have been challenged by some recent observations: cancer stem cells may not necessary to be rare within tumors; cancer stem cells and non-cancer stem cells may undergo reversible phenotypic changes; and the cancer stem cells phenotype can vary substantially between patients. Here the current status and progresses of cancer stem cells theory is illustrated and via providing a panoramic view of cancer therapy, we addressed the recent controversies regarding the feasibility of cancer stem cells targeted anti-cancer therapy.

## A BRIEF VIEW OF ANTICANCER THERAPY

First initiated in 1946, nitrogen mustard was used as a chemotherapeutic agent for cancer therapy [1]. By the early 1990s, anti-cancer drug development had been transformed from a low-budget, Government-supported research effort to a high-stakes, multi-billion dollar industry [2]. This trend continued for the following 20 years. In 2014, it was reported that anticancer drugs accounted for 10.8% of the total market share of the pharmaceutical industry with 100 billion US dollars [3]. In sharp contrast to the rapid development of anticancer drugs, it is reported that cancer has surpassed heart disease to become the number one cause of death worldwide [4]. Even in developed countries such as Australia, cancer mortality rates have not changed significantly during the near 30 years spanning from 1982 to 2011 [5]. The classical cancer theory may underpin this unchanged cancer mortality rates.

## STOCHASTIC CANCER THEORY MAY BE OVERLY SIMPLISTIC

**Figure 1:**
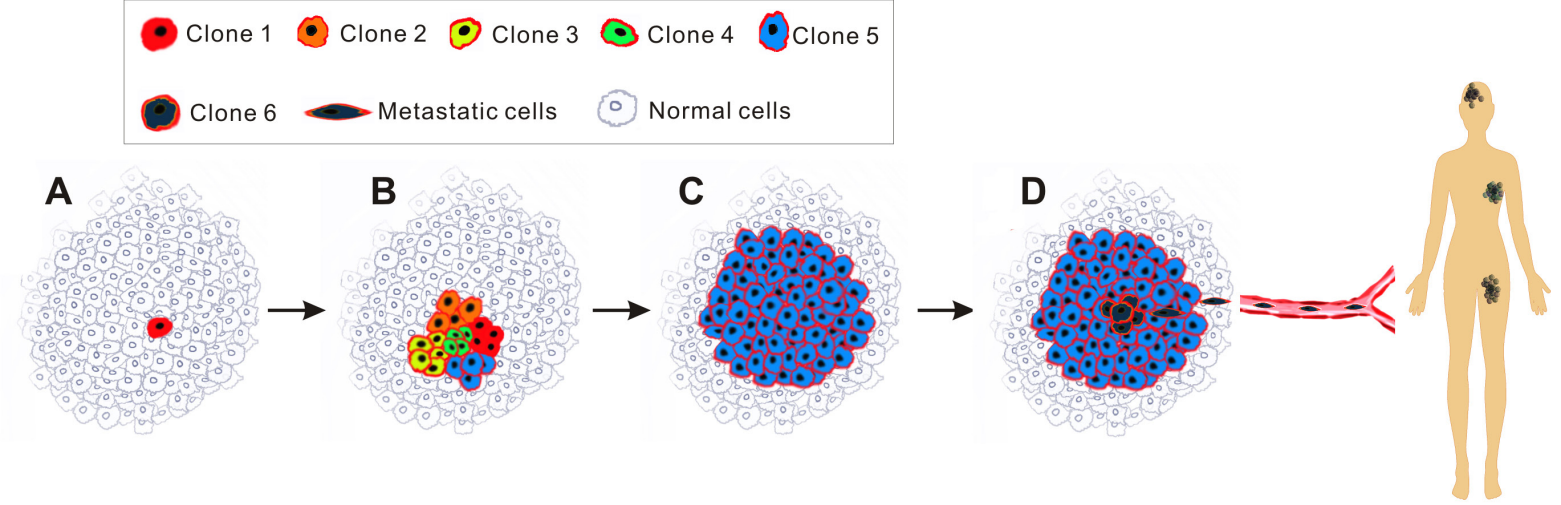
Schematic of clonal evolution model Each cancer cell in tumors harbours similar tumorigenic capacity and the progression of tumour follows the Darwin's theory of evolution. Of note, the red rim of every cancer cell in this diagram illustrates that they all originated from a single cancer cell (red cell in A). **A.**, radiation/carcinogens/viruses-induced mutations in a single normal cell (red) transforms it into a neoplastic cell, conferring selective growth advantages over adjacent normal cells. **B.**, the cancer cell proliferates to produce a cell clone (Clone 1) and at the same time, due to genetic instability, various new clones (Clone 2, 3, 4, 5) are generated. **C.**, those clones that cannot survive selective pressures such as hypoxia, hypoalimentation and chemotherapy are eliminated. Occasionally a colony (Clone 5) acquires survival advantage proceeds and cells from this clone expand to become the predominant population until an even more competitive variant emerges. **D.**, this stepwise evolution continues in response to survival pressures throughout the tumor progression, eventually additional mutations endow a group of new cancer cells (Clone 6) with aggressive phenotype, leading to metastasis.

For decades, anti-cancer therapy has been directed by the clonal evolution (stochastic) theory (Figure 1) [6]. This theory proposes that cancer derives from normal somatic cells which undergo at least five genetic mutations [7] before they possess all of the ten cancer hallmarks such as enhanced proliferation, reduced capacity to undergo apoptosis and inhibition of differentiation [8]. However, this classical theory is far from being satisfactory. First, it is difficult to explain the phenomenon of higher incidence of some cancers in the first few years of human life relative to adult years. And it has been suggested that cancer may not simply be driven by the accumulation of mutation with age [9]. Furthermore, since differentiated somatic cells have a limited life span, it would be theoretically impossible for any given cell to acquire all the necessary mutations [10]. A more reasonable explanation contends that it is likely that the initial mutational hit the cell confers the capacity of unrestrained proliferation, which provides cells with a sufficiently long lifespan to acquire the remaining mutations [11]. Following this logic, it would be reasonable to expect that the status of all cancer cells in a tumor would be similar and in principle, each viable tumor cell is equally capable of forming a new tumor (Figure 1). However, this hypothesis is paradoxical to a well-known phenomenon - usually more than 10,000 cancer cells are required to reproducibly initiate tumors in immunocompromised mice [12, 13]. Recent developments in cancer stem cell (CSC) theory suggest that the classical theory of cancer initiation and progression may be overly simplistic [14].

## A REVOLUTIONARY ANTI-CANCER STRATEGY PROMISED BY CSC THEORY

**Figure 2:**
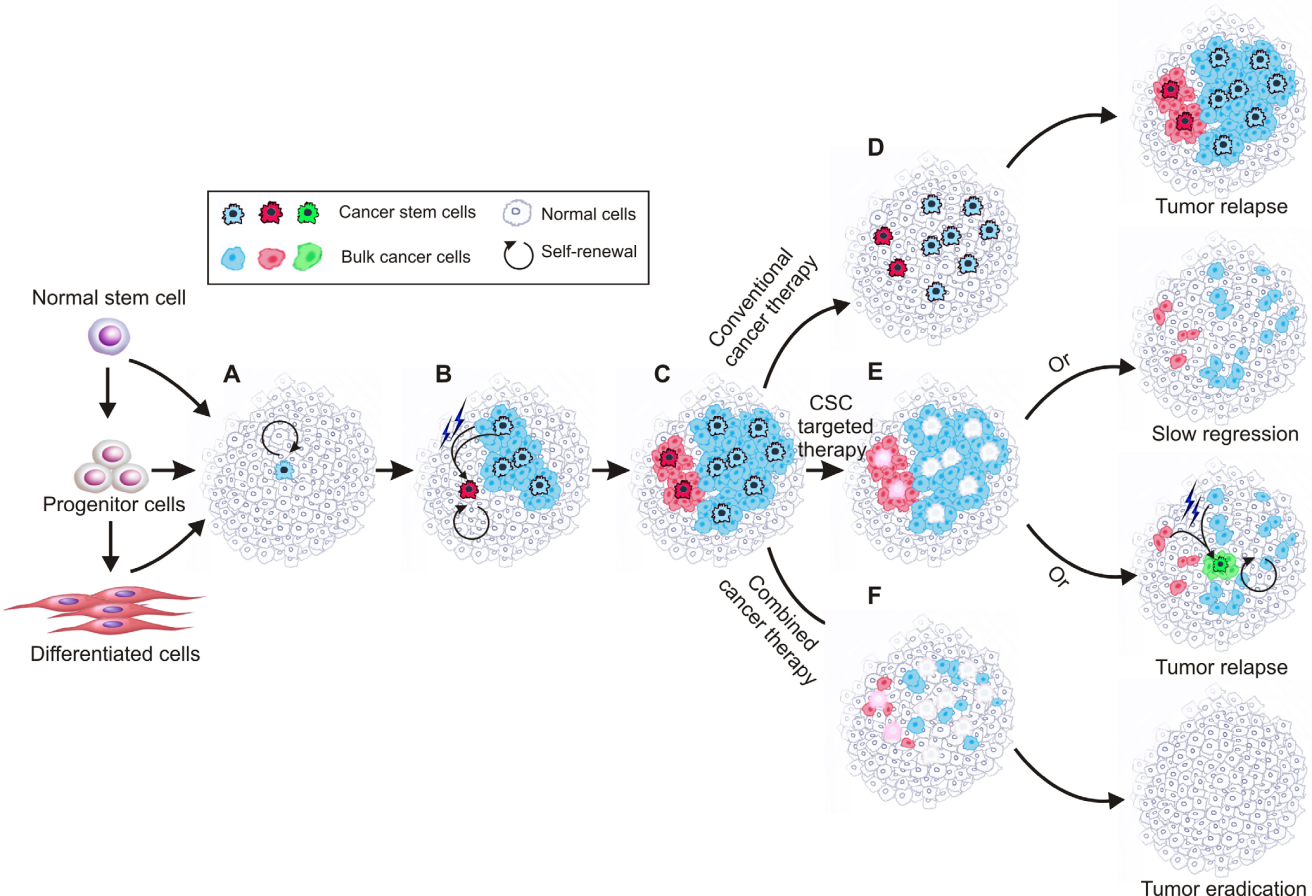
Schematic of current cancer stem cell theory Cancer stem cells are solely capable of self-renewal and unlimited replication and responsible for maintaining the whole tumour. Cancer stem cells show plasticity so that under certain microenvironment, normal cancer cells can convert to cancer stem cells. During tumour progression, different cancer stem cell clones coexist, which are abide by the principle of evolution. **A.**, a cancer stem cell forms due to mutations in normal stem cells, progenitor cells and/or differentiated cells; **B.**, the created cancer stem cell divides asymmetrically and generates daughter cancer stem cells and differentiated bulk cancer cells that can acquire mutations subsequently. At the same time, a new cancer stem cell can be created from mutated cancer stem cell or bulk cancer cell; **C.**, different types of cancer stem cells coexist and are responsible for the observed tumor heterogeneity. **D.**, conventional chemotherapy kills bulk cancer cells but largely leaves chemo-resistant cancer stem cells untouched, leading to tumor relapse. **E.**, killing the cancer stem cells leads to gradual tumour regression, during which new cancer stem cells may converted from mutated bulk cancer cells and cause tumour relapse; F, targeting both cancer stem cells and the bulk cancer cells may result in eventual tumor eradication.

The CSC theory is based on experimental evidence that the status of different cancer cells in a tumor is not equal, similar to that of normal tissues, with some rare undifferentiated CSCs at the top of the hierarchy responsible for maintaining the whole population of cells in a tumor [15]. As shown in Figure 2, these cells share several key properties with normal stem cells [16]. The first such property is self-renewal. CSCs are built to last a lifetime and possess the ability to renew themselves indefinitely with an undifferentiated state. The second property is asymmetric division, which, in addition to self-renewal, is responsible for giving rise to differentiated daughter cells which make up the bulk of the tumor and are characterized by rapid propagation and limited or no proliferative potential in the case of progenitor and bulk cancer cells, respectively. Understanding this phenomenon is important for cancer therapy, as it means that the contribution of these daughter cells to the long-term sustenance of the tumor is negligible [17]. In a tumor, only CSCs are able to initiate tumors as they are solely capable of self-renewal and unlimited replication [18]. Third, CSCs are resistant to electromagnetic and chemical insults. This is mainly because of their infrequent replication [19], heightened activation of DNA repair mechanisms (resulting in a lower apoptotic rate) [20], active drug efflux system [21, 22] and increased defences against reactive oxygen species [23].

The CSC theory is not an entirely new concept, having previously been discussed and investigated for decades [24]. The major reason for it becoming one of the hottest topics in current cancer research [25] lies in the explanation it provides for the poorly understood phenomena observed in both in the clinic and laboratory. From the perspective of the CSC theory, CSCs are the prime sources of tumor recurrence and metastasis, as they confer resistance to traditional electromagnetic and chemical insults by various strategies. The cancer will re-occur months or years after treatment. Thus, most of the metastatic cancers are hardly curable with current anti-cancer treatments (which mainly target the bulk cancer cells), even when the initial response to radiation or chemotherapy is encouragingly robust. And in the laboratory, the rarity of CSCs in a tumor dictates that a huge amount of cancer cells are needed to initiate tumors in animal models. Another reason why the CSC theory has generated such enthusiasm is because of the hope that a new anti-cancer strategy may emerge - aiming not at reducing tumor bulk, but rather at targeting the beating heart of the tumor, the CSCs [26].

## CONTROVERSIES OVER CURRENT CSC THEORY

The CSC theory is possibly the most controversial topic in current biomedical research - it is even hard to reach an absolute consensus on the most basic issue of how to name this group of cells. In recent 10 years various names such as CSC, stem cell-like cancer cell, tumor-initiating cell and tumor-propagating cell have been suggested by different research groups. In fact, this is why in many occasions the CSC theory is also referred to the CSC hypothesis [28]. However, it is understandable considering our understanding of CSCs is still not complete and generally based on the understanding of normal stem cells. Currently, the controversy over the CSC theory focuses mainly on the origin and frequency of CSCs as well as their phenotypic and functional properties [29].

## HOW CAN DORMANT CSCS MAINTAIN A CERTAIN POPULATION SIZE IN TUMORS?

A logically paradoxical concept regarding CSC theory is that CSCs have to be dormant to be resistant to therapy yet have to proliferate together with normal cancer cells to maintain a certain proportion size in tumors. This contradiction has long been explained by the introduction of the concept of “cancer stemloids”. According to this explanation, not all CSCs in tumors are proliferating self-renewing cancer cells. While true CSC is shielded from selective pressure and unable to drive tumor progression, cancer stemloids undergo clonal selection, accumulate mutations and eventually drive tumor progression [27]. Actually, this explanation is theoretically important as it provides a basis to design therapies to selectively kill proliferating self-renewing CSCs without killing normal stem cells. This is because currently reported CSC markers are often expressed on normal stem cells as well. The proliferating yet self-renewal status of cancer stemloids distinguishes them from the quiescent normal stem cells. By targeting stem cell markers only in cycling cells through a combination of stem cell targeted antibodies and anticancer drugs that are toxic only to cycling cells, normal resting stem cells can be spared [30].

## DOES CSC HAVE TO BE RARE?

According to the classical CSC theory, only exceedingly rare CSCs in tumors have the capacity to initiate tumors. For example, a frequency of less than 0.0001% has been reported in acute myelocytic leukaemia (AML) [14, 31, 32]. Surprisingly, some recent research findings suggested that the proportion of stem cell-like cancer cells in a tumor could be as high as one in four [31, 33-35], which challenged one of the basic principles of CSC theory - the hierarchical relationship among cells in a tumor.

Currently, three methods that originally developed for the analysis of adult stem cells including mammosphere assay, cell surface marker expression assay, *in vivo* tumor initiating assay (coupled with limited dilution assay) have been commonly employed for CSC related assessment (see Box 1). Among them, the *in vivo* tumor initiating assay, which involves xenotransplantation of sorted cancer cells (based on specific cell surface markers) into immunodeficient mice [36], has been regarded as the single “gold standard” to define human CSCs. The controversial results regarding the frequency of CSCs may have caused by the different research models and experimental setup employed by different research groups. For example, in the paper “Tumor growth need not be driven by rare cancer stem cells”, Kelly et al. reported that at least 10% of the bulk tumor cells in several transgenic mouse models of leukaemia and lymphoma were capable of initiating malignant growth upon transplantation into mice [33]. However, transplanting mouse tumor cells into histocompatible mice recipients obviously does not meet the “gold standard”(transplanting human cells to immunodeficient mice) and therefore could not speak for human CSCs. In Quintana's experiment [31], human melanoma cells were transplanted into immunodeficient mice. However, instead of employing commonly used NOD/SCID mice, non-obese diabetic, experiments were conducted with severe combined immunodeficient (NOD/SCID) *Il2rg^−/−^* mice.

Undoubtedly, the current *in vivo* tumor initiating models used to assess CSCs is a suboptimal “gold standard” with intrinsic limitations [37]. For example, the mouse tissues to which human cancer cells are transplanted provide a different microenvironment to the original environment from where they arise. In recent years, although improvements to the xenotransplant models have dramatically increased their sensitivity and reliability (see Box 2), it is still accepted that the variations in animal models used for CSC assessment affect the CSC frequency measured quantitatively but not qualitatively [17]. Keeping this in mind, it is unsurprising to see differences in CSC frequency reported among studies in which different animal or cancer cell models had been employed. Since it is ethically impossible to transplant cancer cells to human bodies, this debate will most likely remain unsolved in the near future. The different results in CSC frequency may also result from the heterogeneous feature of tumors. As has been reported, even strictly defined normal tissue stem cells showed different differentiation and self-renewal capacities in accordance with different sites or stages of development [38, 39]. Considering the even higher heterogeneity present among tumors, it is actually expected to see a certain degree of difference in the CSC frequency.

Recently, based on observations that there may be a large proportion of CSCs in tumors, some researchers questioned the necessary of the CSC-targeted anticancer therapy [40]. Obviously, there are flaws with this argument. First, according to the analyses above, the data on CSC frequency itself is affected by different experimental setting and the heterogeneous status of tumor and therefore debatable. Second, it should be emphasized that the fundamental hypothesis underlying the CSC theory is based on the phenomenon of the existence of purified single cells with tumor-initiating capacity rather than the absolute frequency of them [41]. It follows that the frequency of CSCs within a tumor is irrelevant to the concept of whether a tumor adheres to the CSC theory. Even if it is true that therapeutic resistant CSCs make up a large proportion in some types of tumor, the therapeutic implications of CSCs would remain the same and from another perspective, it would only indicate that controling CSCs will be more urgent and more challenging than previously expected.

## THE IMPLICATION OF CONVERSION BETWEEN NON-CSCS AND CSCS?

Early understanding of CSC theory has suggested that CSCs arise from normal stem cells [42]. This is because the majority of cancers develop in epithelia that undergo substantial cell turnover. In epithelial tissues, only stem cells remain in the body and proliferate for long enough to accumulate the number of mutations required to develop into cancer. However, recent studies suggest that the state of CSCs is quite plastic, such that they can arise from a progenitor or even normal cancer cell that has acquired the capacity for sustained self-renewal through mutation, epigenetic change, or both [24, 37, 43, 44]. Indeed, this plasticity has been demonstrated in human colon cancer cells by simply retrovirally introducing a set of defined factors (OCT3/4, SOX2 and KLF4) [45]. This observed plasticity of CSCs challenged another basic hypothesis of CSC theory - unidirectional development, and raised the question of “how can a CSC truly be a stem cell if non-CSCs can become CSCs? [29]”

In fact, this phenomenon is not exclusively observed in CSCs. As reported several times, under certain conditions, differentiated epithelia tissues including skin, mammary gland and intestine could display regenerative activities [46, 47] - a main property of stem cells. Notably, the 2012 Nobel Prize has been awarded to investiga­tors who demonstrated that mature, specialized cells can be reprogrammed to become immature cells capable of developing into all tissues of the body [48, 49]. Considering the great impacts of hypoxia [50], acidic stress [51] and nutrient deprivation [52] on tumor microenvironment, it should come as no surprise to see a certain extent of plasticity between CSCs and bulk cancer cells.

Given the potential plasticity of CSCs, it has been contended that “only if the CSC phenotype is a stable trait, will it be advantageous to selectively target CSCs as a cancer treatment” [17]. Certainly, the plasticity of the CSC state adds complexity to both CSC regulation and cancer in general. However, from the perspective of cancer therapy, what's more important is to verify if CSCs exist and if they are the root of tumor recurrence and metastasis. In contrast, it is not that important as to where CSCs come from. If there is anything to be learned, it is that both CSCs and the bulk cancer cells should be targeted to cure cancer (Figure 2) [53]. Actually, this is exactly why almost all of the current clinical trials aimed at CSCs are combined with traditional tumor treatment [19].

## ARE CSC MARKERS RELIABLE?

CSC markers are cell surface proteins associated closely with specific phenotypic and transcriptional profiles of CSCs [54]. In recent years, with various CSC markers being reported in various types of cancers, CSC markers hold great potential in not only clinical diagnosis and basic cancer research but also in developing CSC targeted anti-tumor therapies[55, 56], as detailed in recent reviews [25, 57].

However, it should be noted that thus far there is no uni­versal marker for CSCs identified. All of the currently described CSC markers can be detected not only on CSCs but also, more or less, on normal stem cells or normal cancer cells or even normal tissues [25, 57, 58], leading concerns of “The markers that have been used so far to define CSCs constitute unlikely candidates for antibody therapy given that they are usually broadly expressed in healthy tissue” [16] and “relying on markers will fool you [29]”. These comments imply that “CSC markers should be detected only on CSCs” and “there should be a CSC marker expressed on many types of CSCs”. In reality, since current understanding suggests CSCs probably originate from either normal stem cells or bulk cancer cells, it is conceivable that CSCs share certain degree of protein expression pattern with the cells they come from. Moreover, considering the extensive heterogeneity even in a single tumor, it is unrealistic to expect a marker to be observed on many kinds of CSCs. Different CSC may have different CSC markers. However, once a marker can be confirmed to be overly expressed on CSCs, such a marker can be exploited for targeted cancer therapy, even if it is only expressed on one type of CSC, or it is also expressed at a low level in other tissues [57].

Compared with the specificity of CSC markers, the stability of CSC markers represent an even bigger obstacle for CSC diagnosis and treatment. Recently, it has been reported that the cell populations (defined by surface marker/marker combination) meeting the gold standard of CSC assessment (*in vivo* tumor initiating assay) has not proved to be singular or even stable [59]. For instance, in earlier studies it was recorded that the AML CSCs were confined in CD34^+^CD38^−^ population as confirmed through *in vivo* tumor initiating assay. However, subsequent experiments observed that CD34^+^CD38^+^ AML cells also demonstrated similar CSC activity [60-62]. In other cases, similar phenomena of coexisting or unstable CSC markers have also been observed in several of human solid tumors [63-66] and human acute lymphoid leukaemia (ALL) [67]. The instability of CSC markers may have resulted from the well-established notion that the malignant tumor cells with aberrant gene expression regulation are capable of altering developmental control and/or the stability of the expression of cell surface markers. This is especially true when studies were conducted *in vitro* [68].

Taken together, while CSC markers are informative to understand the population being studied and promising for active targeting, they alone cannot define CSCs [29]. Given the current lack of specificity and instability in certain cases, the reliability of any CSC marker in specific application settings (CSC analysis or targeted treatment) has to be tested experimentally *via* the *in vivo* tumor-initiating assay.

**Figure 3:**
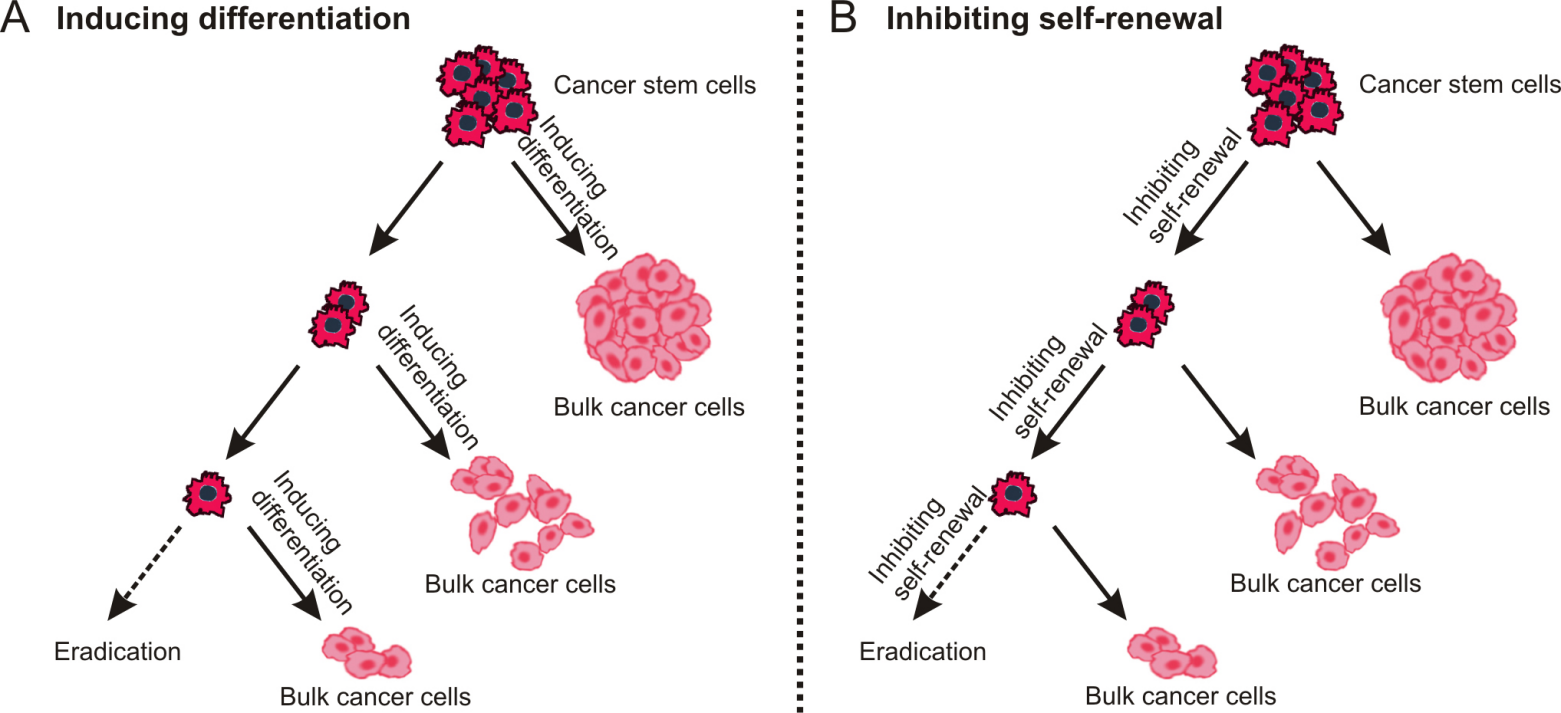
Destemming cancer stem cells The inhibition of self-renewal and inducing differentiation may lead to similar outcome - fewer CSCs and more normal cancer cells are generated during asymmetry division.

## STRATEGIES FOR CSC TARGETING

As hoped, a win in the clinic will solve many of the controversies regarding the CSC theory [19]. In recent years, despite expensive failures in earlier clinical trials and fundamental discrepancies about CSC theory, a new round of “gambling” has been launched, with more than sixty CSC-targeted reagents currently being registered for clinical trials [19]. For CSC therapy, the enhanced drug-resistance and microenvironment (niche) of CSCs represent feasible targets and have been intensely exploited. Our knowledge of tumor genetic and signalling pathways collected in the past decades including the increased understanding of various oncogenic derivatives, adhesion molecules, antibody-accessible surface components, signalling intermediates, survival pathway elements, chromatin modifiers and metabolic targets provides valuable tools and targets in this area [69-75]. Generally, CSC targeted therapies can be classified according to the therapeutic strategies employed as detailed below.

## “DESTEMMING” CSCS

Although the strategy of “destemming” CSCs [76] includes two aspects, either promoting CSC differentiation into non-CSCs or inhibiting their self-renewal property, the ultimate aim is the same - “exhausting dormant CSCs” (Figure 3).

With mounting evidence suggesting that there are similarities between normal stem cells and CSCs in terms of their self-renewal and differentiation signaling pathways [77-79], several critical signaling pathways involved in self-renewal and differentiation of normal stem cells have been studied intensively.

By far the most exploited signaling pathways associated with the self-renewal of CSCs are the Hedgehog signalling, Notch signalling and Wnt/β-catenin signalling pathways, [77, 80]. Several agents targeting these pathways have shown promising preclinical results and are currently under investigation in phase I and II clinical trials [19, 81]. Actually, Vismodegib, a Hedgehog inhibitor approved for basal cell carcinoma treatment has made its way into clinic in 2012 [82]. Targeting Notch signaling pathway, a pathway best known for shaping embryonic development, also demonstrated potential in regulating CSC fate in various types of cancers, including both solid tumors and leukaemia [72]. Indeed, different Notch inhibitors such as γ-secretase inhibitors and monoclonal antibodies have been evaluated in the past few years [72, 83-85]. In 2014, OncoMed's Tarextumab, a Notch pathway targeted monoclonal antibody attracted attention, in a safety study for pancreatic cancer - a disease in which traditional chemotherapy rarely helps, the combinatorial treatment of Tarextumab and conventional chemotherapeutic drugs resulted in the stabilization or shrinkage of the tumor over periods of as long as 12 months in 83% of 29 patients [86]. At present, a phase II trials has been commenced for Tarextumab in pancreatic and lung cancers [19].

As for promoting the differentiation of CSCs, bone morphogenic protein (BMP) and oncostatin M (OSM) are among the mostly studied signalling pathways. Encouraging results have been reported recently. For example, through the stimulation of BMP signalling in colorectal CSCs by BMP4 (a natural ligand of MBP receptor), Lombardo et al. observed not only increased terminal differentiation but also enhanced chemo-sensitivity of CSCs [87]. The phenomenon of chemo-sensitization was also detected following the activation of OSM signalling in breast [88-90] and liver CSCs [91]. All of these results indicate that the combinatorial treatment of signal transduction and conventional chemotherapy may aid in eradicating CSCs [90]. Recently, the importance of phosphatidylinositol 3-kinase/Akt/mammalian target of rapamycin (P13K/mTOR) signalling pathway in regulating the balance between proliferation and differentiation of CSCs was revealed [92]. Some inhibitors targeting this pathway have been showing promise in CSC targeted therapy, with some dual inhibitors undergoing clinical trials with advanced breast, ovarian and small-cell lung cancers [92].

What should be taken into account is that since these signaling pathways are shared by both CSCs and normal stem cells, and these pathways auto-regulate and interact with many other pathways, any global adjustment of these pathways will likely disturb the function of normal stem cells and cause potential toxicity. For example, in the late 2000s, the U.S. National Cancer Institute together with commercial partners conducted small-scale safety trials of reagents aimed at CSC signaling pathways (including Hedgehog and Notch) and observed serious side effects on normal stem cells [19]. Therefore, considerable caution must be exercised when evaluate the full effects of intervention with any single pathway [93].

## DIRECTLY TARGETING DRUG RESISTANCE MECHANISMS OF CSCS

CSCs are best characterized by enhanced drug-resistance, which could be derived either directly from their previous generations or through accumulation of the constant genomic and epigenetic mutations [94]. While both promoting differentiation and inhibiting self-renewal can destem CSCs and eventually increase the chemo-sensitivity of CSCs, molecules or pathways directly related to drug resistance of CSCs such as multidrug resistance proteins and anti-apoptotic pathways have also been explored.

Accumulating evidence suggests that some protecting mechanisms of normal SCs such as MDR transporters also operate in CSCs. These transporters, belonging to ATP-binding cassette (ABC) family, are well-known to be able to pump exogenous small molecules out of cell membrane and therefore cause resistance to a wide range of conventional drugs. Furthermore, some transporters such as ABCB5 has been used as CSC marker for melanoma CSCs [95]. In fact, the overexpression of ABCB2, also known as breast cancer resistance protein (BRCP1), was recently shown to be responsible for chemo-resistance of glioblastoma CSCs to a variety of agents including Paclitaxel, Carboplatin, Etoposide, and Temozolomide [96]. However, the role of these drug efflux pumps in modulating drug resistance of CSCs has been challenged based on the fact that despite considerable efforts, rare clinical benefit of inhibitors to such proteins has been realized [97], implicating a mechanism of redundancy and/or complexity in this area.

Although the active survival pathways have not been characterized in detail in CSCs, the deregulation of both extrinsic and intrinsic apoptotic signaling pathways have been reported in this population of cells [98]. For example, the overexpression of the Bcl-2 family, a group of anti-apoptotic proteins related to the critical step of intrinsic apoptotic cascade (mitochondrial outer membrane permeabilization) have been observed in most types of CSCs [99, 100]. Accordingly, Bcl-2 inhibitors such as ABT-199, ABT-737 and TW-37 have shown prominent CSC targeting capacity. According to a recent report, as a single agent, ABT-737 alone was able to inhibit the frequency of CSCs and reduce CSCs content in treated acute myeloid leukaemia (AML) as well as solid tumors such as lung and breast cancers [100-103]. On the other hand, targeting extrinsic apoptosis pathway, especially TNF-related apoptosis-inducing ligand (TRAIL), is also showing promising results [104]. In addition to directly using TRAIL as a drug, engineering of mesenchymal stem cells (MSCs) for TRAIL delivery represents a novel therapeutic option. After systemic injection, TRAIL-expressing MSCs was observed to be able to localize to the site of the tumor and successfully eliminate metastatic CSCs [98, 105].

As another important aspect of apoptotic machinery, the inhibitor of apoptosis protein (IAP) family has been regarded as the last protective measure against apoptosis since it prevent both intrinsic and extrinsic apoptosis by inhibiting caspase activity [106]. Among the eight human homologues of IAP family, survivin and XIAP have received more attention in recent years, with more than 30 survivin- and XIAP-based anti-cancer preparations undergoing clinical trials [107]. From the perspective of CSC targeted therapy, survivin is quite unique. First, different from other IAP family members and Bcl-2 family members, survivin specifically overexpresses in human cancers and dose not express in most adult tissues, which makes it an attractive target for anticancer therapy [106]; second, together with Hiwi, hTERT genes, survivin has been proposed to be an important stem cell-associated gene and the co-expression of all of these three genes has been shown to result in a significantly increased risk of tumor-related death in patients with soft-tissue sarcoma [108]; last, enrichment of survivin has been described in different types CSCs including AML, glioblastoma, liver, breast and astrocytoma. *Via* suppression of survivin, prominent induction of apoptosis of CSCs was observed in breast and liver cancer as well as in recurrent glioblastoma [109-113].

## TARGETING THE CSC NICHE

The concept of the CSC niche is derived from the understanding of the normal stem cell niche, in which normal stem cells have discrete locations in tissues and are regulated by its microenvironment [114]. Similarly, CSCs in tumors are in a complicated ecosystem consisting of bulk cancer cells, various endothelial, hematopoietic, stromal fibroblast and perivascular/vascular cells. As a component of this system, the CSCs are heavily influenced and supported by their surrounding environment [115]. In fact, the overall fitness of any cell (CSC/non-CSC) in a tumor is modulated by its microenvironment. This is because the interaction of the tumor components inevitably causes metabolic inconsistency within a tumor and results in topical nutrient deprivation, hypoxia or other survival pressure [50-52]. These survival stress in turn pushes all the surrounding cells towards a status best fitting its particular microenvironment and eventually creates the well-known heterogeneous property of tumors [115]. Specifically for CSCs, the frequently observed discrepancies in drug sensitivity between *in vitro* and *in vivo* treatments provide evidence that the niche in which a CSC is located pivotally determines its response to a given treatment [116]. And when the cell phenotype was studied, it was discovered that the epithelial to mesenchymal transition (EMT) of CSCs, which usually results in more aggressive and metastatic phenotype, was affected considerably by their niche [117]. The implication of all these is that the niche of CSCs directly affects the drug sensitivity and mobilization of CSCs and therefore represents a potential target for CSC-directed therapy.

In recently years, the influences of adhesion receptors, cytokine receptors, membrane-bound cytokine ligands, and various chemotactic factors upon the status of CSCs have been studied [114]. These results, along with the previously described cellular components of CSC niche such as endosteum cells in the bone marrow, perivascular/vascular cells and tissue macrophages [118], provide us with precious opportunities to develop CSC niche-targeted therapies. Among them, focal adhesion kinase (FAK) is one of the mostly investigated targets in both academia and industry [119, 120]. Also known as protein tyrosine kinase 2 (PTK2), FAK is an enzyme that plays an important role in cell adhesion, spread, motility, invasion, metastasis, survival, angiogenesis, and EMT. Many believe that blocking FAK could not only directly eradicate CSCs but also prevent these rare cells within primary tumors to travel through the bloodstream and seed metastases [19]. Several orally available FAK inhibitors such as VS-6063 and VS-4718 have shown promise in counteracting CSCs in recent clinical trials [121]. Another promising targets is CXCR4, which is expressed on many types of cancer cells and works as a receptor for stromal cell-derived factor 1 (SDF1; also termed CXCL12). As a niche-derived chemo attractant for CXCR4^+^ cells, SDF1 is able to enhance the entry of CXCR4^+^ cells into the bone marrow [122]. Recently, several effective CXCR4 antagonists have been developed to immobilize CSCs and sensitize them to traditional chemotherapies [123, 124], with Plerixafor (AMD3100) and some T14003 analogs being tested in clinical trials for leukaemia [122].

Pioneered by Judah Folkman back in 1971 [125], targeting of angiogenesis has long been a hot point in cancer research. However, the benefit of targeting angiogenesis upon inhibiting CSCs was studied just recently. Encouraging results were collected from earlier studies designed to explore the CSC targeting capacity of clinically available antiangiogenic drugs such as Bevacizumab, Sunitinib, and Lenalidomide. For example, *via* treating U87 glioma bearing mice with bevacizumab, Calabrese et al. observed decreased microvasculature density and tumor growth, in addition, the authors observed a reduction in the number of CD133^+^/nestin^+^ CSCs [126]. At the same time, very similar results on glioblastoma were also observed by other investigators [127]. But the hope of employing ready-made antiangiogenic drugs to deal with CSCs was shattered when accumulating clinical and preclinical evidence indicated that the benefits of antiangiogenic agents to the long-term overall survival of patients was negligible [128, 129]. Furthermore, new research using preclinical models suggest that antiangiogenic agents actually increase invasive and metastatic properties of cancer cells and even worse, both Sunitinib and Bevacizumab, two of the most frequently used antiangiogenic agents, adversely increased the population of CSCs in malignant tumors [130]. In light of these limitations, the approval of Bevacizumab for treatment of advanced breast cancer has recently been revoked by U.S. FDA [131]. These adverse effects are understandable. By inhibiting the growth of new tumor vasculatures, the harsh environment (hypoxia and hypoalimentation) created by antiangiogenic agents pushes the relevant cancer cells/CSCs down towards an extreme path - death or evolve into a more malignant state. With the activation of critical molecules for CSC survival such as hypoxia-inducible factor 1α and Akt/β-catenin regulatory pathway [130], these antiangiogenic agents in fact create a microenvironment in which the survival advantage of CSCs was enhanced. Therefore, it is now suggested that angiogenesis-targeted treatment alone may not be sufficient to improve patient outcome. Rather, it is imperative to combine antiangiogenic agents with CSC targeted treatments [130].

However promising it may be, CSC niche associated studies do not come without concerns. First, it is still unclear how particular cells in the CSC niche contribute to the behaviour of CSCs and how their influence on CSCs are mediated at a molecular level [132]; second, further studies are needed to investigate whether, and to what extent, CSCs contribute to important features of their microenvironment through autocrine or paracrine mechanisms, or by creating clonal niche components [133]; last, similar to the strategy of targeting destemming signaling pathways, CSCs share similar niches with normal stem cells, and therefore potential side effects associated with targeting CSC niche have to be considered and circumvented [134].

## CONCLUSION

It has never been easy to cure diseases such as cancer. Over the past 60 years, too many inspiring discoveries and techniques for cancer treatment have eventually been shown to be relatively less useful in the clinic [19]. Admittedly, the current CSC theory remains contentious and the controversies may remain in the next few years. However, CSC-targeted therapy does provide us with a new and promising opportunity to treat tumor cells that are resistant to current therapies and responsible for recurrence and treatment failure. Furthermore, the concept of CSC-targeted therapy is feasible as evidenced by many of the encouraging results obtained in recent CSC-related clinical trials. With better understanding of the fundamental biology of CSCs, improved functional assessment models and achievements in biotechnology such as gene expression profiling, next generation sequencing and high content screening, we are closer to achieving the goal of eradicating CSCs.

## BOX 1

### Current CSC assessment models

Because of the similarities between CSCs and normal stem cells in their primary characteristics (self-renewal and multipotent differentiation), methods developed originally for analysis and characterization of adult stem cells have been transferred to CSCs. The *in vivo* tumor initiating assay is by far the single gold standard for CSC analysis. This approach involves demonstrating the tumor initiating capacity of cells that are directly isolated from tumors to produce new tumors in immunocompromised mice. It was firstly conducted to enumerate CSCs in ALL [135], AML [136] and chronic myeloid leukaemia (CML) [137]. Later, its application was extended to solid human tumors including breast cancer [13], colon cancer [138, 139], ovarian cancer [66], lung cancer [140] and head and neck cancer [141]. However, the *in vivo* tumor initiating assay is not only expensive but also time consuming, with a standard assessment taking as long as 6 months or even longer. Therefore, a reliable *in vitro* assay model is required to efficiently and cost-effectively define CSCs. In 1992, Reynolds and colleagues developed an *in vitro* technique termed the neurosphere assay to quantify activity of neural stem cells [142], which provides the basis for the most popular *in vitro* CSC assay - mammosphere or tumorsphere forming assay [143]. Recently, this assay has been commonly employed in various CSC-associated studies and often serves as a surrogate for the *in vivo* tumor initiating assay. The tumorsphere forming assay involves the dissociation of cultured cells or tumours into a single cell suspension and subsequent culture on non-adherent substrata in the presence of serum-free media supporting the growth of CSCs until they form organized cellular spheres, each containing at least 50 cells. Of note, since progenitor cells are able to proliferate several times, the formation of primary tumorspheres is in fact the measure of a collective activity of CSCs and progenitor cells. Therefore, to accurately evaluate CSCs, primary tumorspheres should be harvested, dissociated into single cells, and passaged to create a ‘second’ generation or even tertiary tumorsphere to exhaust the self-renewal capacity of progenitor cells [144]. Another method for CSC assessment is based on the specific phenotypic and transcriptional marker profiles of CSCs [54]. In practice, this method is very straightforward and cost-effective and therefore more clinically applicable. In recent years, with the increasing number of CSC related cell surface markers reported in various types of cancers, this method holds great potential in not only clinical diagnosis and basic cancer research but also in developing CSC-targeted anti-cancer therapies.

## BOX 2

### Limitations and progression of CSC assessment models

Although cell surface marker analysis represents a convenient CSC assessment method, its reliability relies on a prerequisite that the employed CSC markers must show sufficient stability, generality and specificity. However, considering the phenotypic and genomic heterogeneity shown in tumors even with a similar histological appearance and grade, it is inevitable to see unstable cell surface marker expression patterns among CSCs [25, 57]. As a result, in the past decade, although various surface marker combinations (rather than a single marker) have been successfully used to detect or isolate CSCs in various types of tumors, to standardize this method in clinical application is still a considerable challenge [57].

Compared with surface marker analysis, tumorsphere formation assay is comparatively more reliable. However, concerns regarding this method has been raised given the fact that it is after all conducted in an artificial and less physiological *in vitro* setting. A typical test period for tumorsphere assay lasts one to several weeks, during which the tested cells are likely to undergo abnormal differentiation and transform into a clinically irrelevant state [133]. In addition, the artificial cell culture conditions could unavoidably cause no growth of the tested cells [145]. Consequently, it is widely accepted that the tumorsphere forming assay, although shows comparative advantages in *in vitro* tests, itself is not sufficient for deducing clinically meaningful predictions [143]. For solid evidence of the presence of CSCs, the functional *in vivo* tumor-initiating assay is irreplaceable.

As for the *in vivo* tumor-initiating assay, although immunocompromised mice have been commonly employed, to what extent the results collected from mice faithfully reflect the CSC properties of cancer cells in patients is unclear [94]. First, the relatively shorter lifespan of mice poses the question of how faithfully the results collected from mouse models reflect the clinical outcomes. Applying secondary recipients or long lifespan animal models represent potential solutions for this problem [94]. Second, the altered setting of transplanted tumor cells, including the species difference and the changed microenvironment weigh heavily against the reliability of this assay both phenotypically and genetically [114]. In this respect, the application of genetically modified humanized mouse models has provided a solution to at least partly solve this problem [146-150]. Apart from using modified animal models, orthotopic injection of cancer cells into the targeted organs and supplementing human stromal elements are also beneficial and these have become common practice in recent years [41, 151]. Third, the absence of immune-surveillance in the immunocompromised mouse model compromises its reliability in mimicking the normal *in vivo* environment. Accordingly, the mimicry of natural immune surveillance mechanisms can be partly achieved through injection of specific immune effector cells [152]. Cells used for *in vivo* tumor initiating assay add another layer of complication. Of note, instead of using cells directly derived from patients, cell lines have been frequently used in CSC studies. Although these cell line-based results are commonly translated to the types of cancers they dissociated, the extent to which the behaviour of such cell lines reflecting the clinical tumor cells is highly debatable. To solve this problem, patient-derived primary cells have been confirmed to be an ideal choice. However, it should be noticed that since the *in vitro* cell culture system provides cells with a dramatically different microenvironment from the original tumors from which they derived, the primary cells should not be continuously cultured *in vitro* and amplification of these cells *via* xenograft can improve the reliability of this gold standard assay [29].

A recent study led by Jacobsen and coworkers illustrated a genetic analysis-based novel CSC assessment method to directly analyse CSCs in the human body. Through backtracking of all identified somatic genetic lesions in the bulk bone marrow, the existence of rare and distinct human CSCs was confirmed in myelodysplastic syndrome patients [37]. This study, though elaborate, not only provided direct evidence of the existence of rare CSCs but also provided a genius strategy to bypass the ethical barrier of transplanting cancer cells into humans.
